# Artificial intelligence-assisted identification and quantification of osteoclasts

**DOI:** 10.1016/j.mex.2021.101272

**Published:** 2021-02-18

**Authors:** Thomas Emmanuel, Annemarie Brüel, Jesper Skovhus Thomsen, Torben Steiniche, Mikkel Bo Brent

**Affiliations:** a Department of Dermatology, Aarhus University Hospital, Denmark; b Department of Biomedicine, Aarhus University, Denmark; c Department of Pathology, Aarhus University Hospital, Denmark

**Keywords:** Osteoclasts, Bone histomorphometry, AI-assisted image processing

## Abstract

Quantification of osteoclasts to assess bone resorption is a time-consuming and tedious process. Since the inception of bone histomorphometry and manual counting of osteoclasts using bright-field microscopy, several approaches have been proposed to accelerate the counting process using both free and commercially available software. However, most of the present alternatives depend on manual or semi-automatic color segmentation and do not take advantage of artificial intelligence (AI). The present study directly compare estimates of osteoclast-covered surfaces (Oc.S/BS) obtained by the conventional manual method using a bright-field microscope to that obtained by a new AI-assisted method. We present a detailed step-by-step guide for the AI-based method. Tibiae from Wistar rats were either enzymatically stained for TRAP or immunostained for cathepsin K to identify osteoclasts. We found that estimation of Oc.S/BS by the new AI-assisted method was considerably less time-consuming, while still providing similar results to the conventional manual method. In addition, the retrainable AI-module used in the present study allows for fully automated overnight batch processing of multiple annotated sections.

• Bone histomorphometry
• AI-assisted osteoclast identification
• TRAP and cathepsin K

## Graphical Abstract

**Figure:**
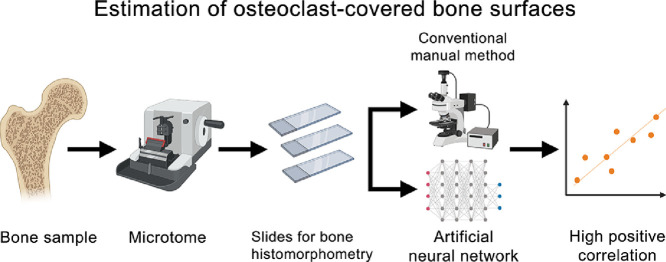

Specifications table

**Table:** 

Subject Area:	Medicine and Dentistry
More specific subject area:	Bone histomorphometry
Method name:	Artificial intelligence-assisted identification and quantification of osteoclasts
Name and reference of original method:	N/A
Resource availability:	Visiopharm VIS software including AI-module is available from https://visiopharm.com/

## Background

Osteoclasts are specialized bone resorptive cells, which are recognized as giant multinucleated cells residing on the bone surface. The osteoclasts adhere to the bone surface by matrix adhesion proteins forming a tight sealing zone thereby creating an isolated sub-osteoclastic space [1]. During bone resorption, the basal osteoclastic cell membrane forms a highly convoluted ruffled border, which contact the bone surface. The ruffled border serves as an exit site for protons, lysosomal proteases like cathepsin K, and phosphatases like tartrate-resistant acid phosphatase (TRAP) [2]. TRAP is mainly stored in intracellular vesicles and vacuoles, making it a favored target for enzymatic histochemistry for identification of osteoclasts. Enzymatic intramolecular rearrangement can be used to detect TRAP by staining with pararosaniline base, where an initial colorless soluble substrate is hydrolyzed and rearranged to an insoluble colored product [3]. Cathepsin K cleaves collagen type I in the bone matrix, and therefore another commonly used approach to visualize osteoclasts is by use of anti-cathepsin K antibodies. Osteoclast identification and quantification are important for ascertaining bone resorption, but the process can be very labor-intensive, time-consuming, expensive, and challenging. Although various image-processing programs have been developed to ease the process, they are still largely dependent on manual inputs and color segmentation [4], [5], [6]. However, the advancement of computational power and artificial intelligence (AI) can be utilized to speed up repetitive tasks like osteoclast detection through AI-based image analysis and workflow standardization. We present the first detailed step-by-step protocol to determine osteoclast-covered bone surfaces (Oc.S/BS) using AI and compare the results directly with those obtained by the conventional manual bone histomorphometric method.

## Method details

We analyzed longitudinal sections of tibial bone either stained for TRAP by enzymatic histochemistry or stained for cathepsin K by immunohistochemistry to determine osteoclasts-covered bone surfaces using AI.

Materials used for AI-assisted identification and quantification of osteoclasts

• Visiopharm VIS with AI module (v. 2019.12 or newer; Visiopharm, Hørsholm, Denmark).
• Pararosaniline base (P7632; Sigma-Aldrich, St. Louis, MO, USA).
• Polyclonal rabbit anti-cathepsin K antibody (AP08851PU-N), 1:200, Acris Antibodies, San Diego, CA, USA) or similar (see the online Supplementary Material for alternatives).
• Slide scanner (NanoZoomer 2.0-HT; Hamamatsu Photonics K.K., Hamamatsu City, Japan).

### Animals, sample preparation and image acquisition

Female Wistar rats from two previously conducted animal experiments were used in this study. The rats were sacrificed by an overdose of 200 mg/kg pentobarbital injected intraperitoneal (Mebumal, SAD, Copenhagen, Denmark) while under general anesthesia administered via inhalation of 4% isoflurane (IsoFlo Vet, Orion Pharma Animal Health, Nivå, Denmark). The right tibiae were immediately removed and immersion-fixed in 0.1 M sodium phosphate-buffered 4% formaldehyde, pH 7.0, for 48 h and then transferred to 70% ethanol.

**Fig. 1:**
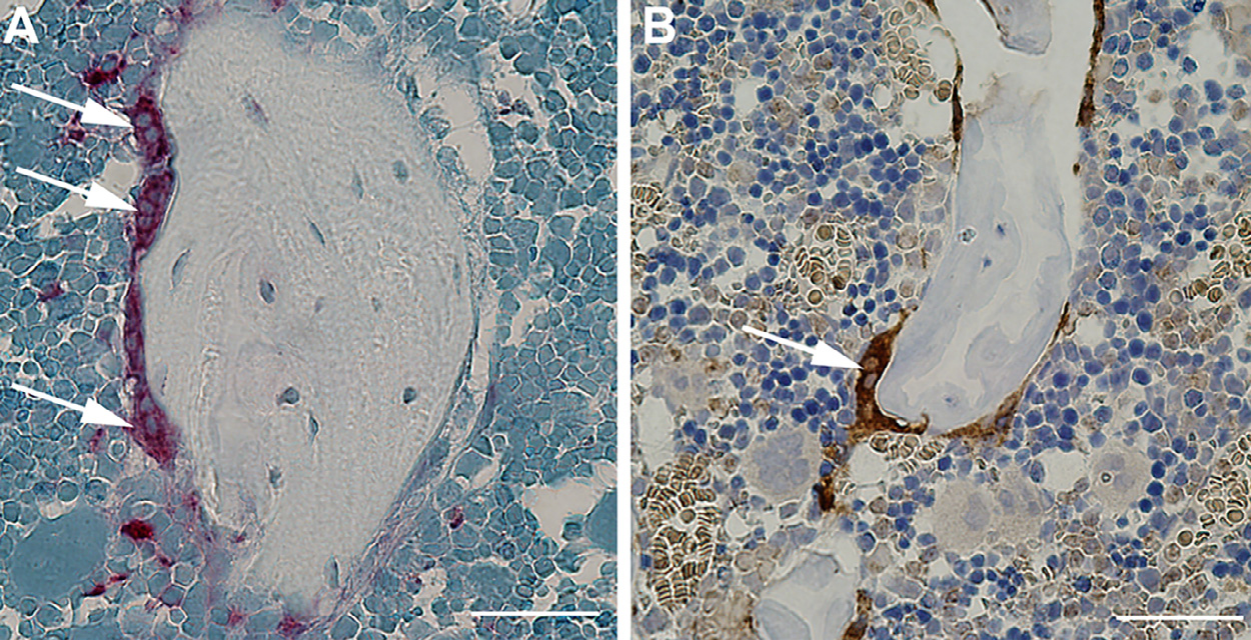
A) Osteoclasts stained for TRAP. Bar = 50 µm. B) Osteoclast stained for cathepsin K. Bar = 50 µm.

The first experiment comprised proximal tibiae from ten 20-week-old rats, which were embedded undecalcified in methyl methacrylate (MMA) and cut into 7-μm-thick sections using a microtome (Jung RM2065; Leica Instruments, Nussloch, Germany). The undecalcified sections were stained for TRAP using pararosaniline base and counterstained with aniline blue (Fig. 1A) using a modified protocol adapted from van 't Hof et al. [4]. The other experiment comprised proximal tibiae from ten 18–20-week-old rats. The bone samples were decalcified in formic acid before being embedded in paraffin and cut into 7-μm-thick sections using the microtome. The decalcified sections were stained for cathepsin K using a primary antibody (polyclonal rabbit anti-cathepsin K antibody) for osteoclast identification (Fig. 1B). The protocols for MMA, TRAP, and anti-cathepsin K antibody are available online as Supplemental Material.

The sections were either projected to a computer monitor as live images by a bright-field microscope (Nikon Eclipse i80, Tokyo, Japan) equipped with a microscope camera (Olympus DP70, Tokyo, Japan) for manual osteoclast identification or scanned with a Hamamatsu digital slide scanner (NanoZoomer 2.0-HT; Hamamatsu Photonics K.K., Hamamatsu City, Japan) for AI-assisted osteoclast identification. In both cases, the Visiopharm software VIS was used for image analysis.

**Fig. 2:**
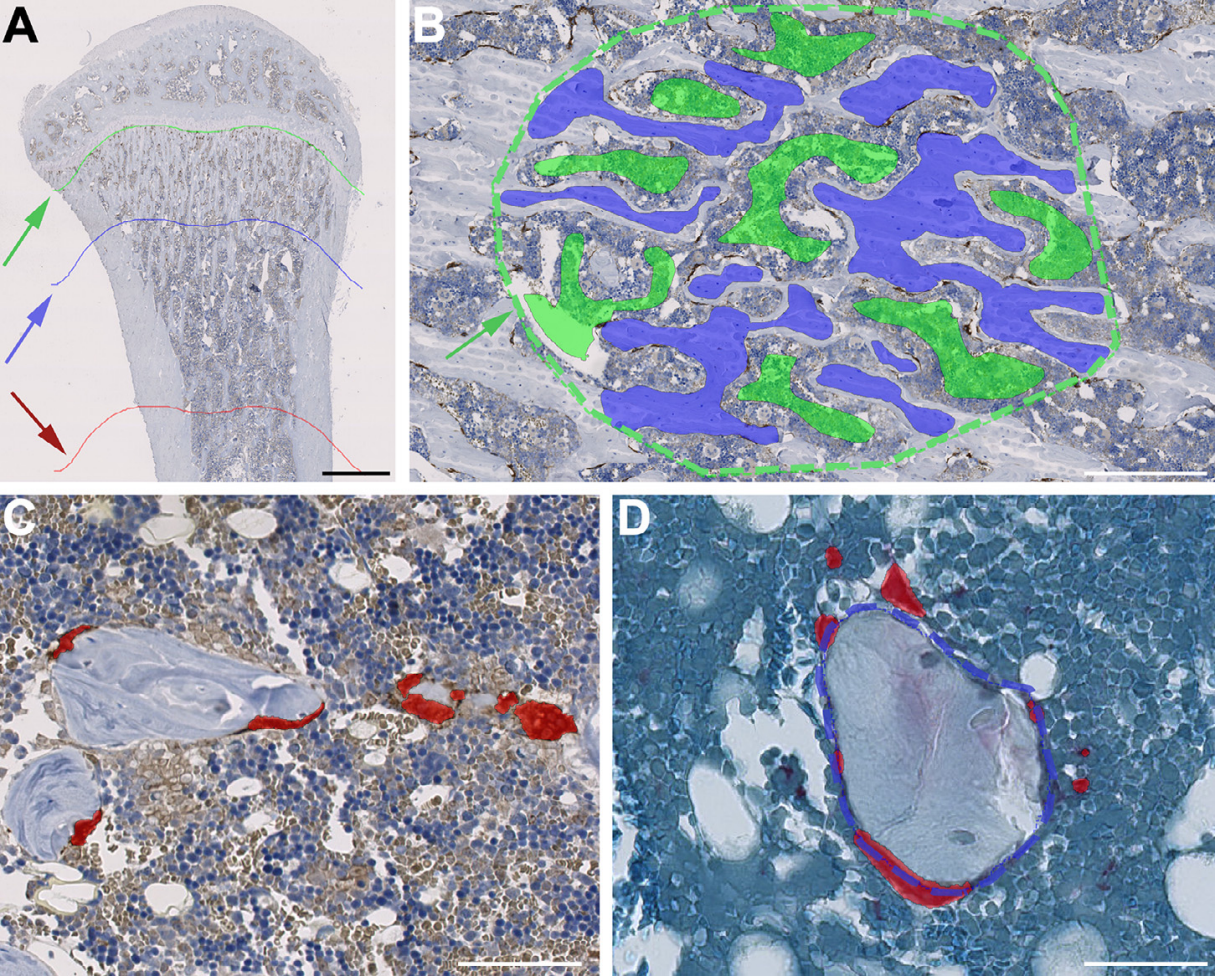
Workflow of AI-assisted identification and counting of osteoclasts. A) Annotation of region of interest (ROI). Green arrow: Annotated line at the distal part of the growth plate. Blue arrow: Annotation line 1500 µm below the growth plate. Red arow: Annotation line 4500 µm below the growth plate. Bar = 1 mm. B) An example of algorithm training. The dotted green circumferential line (green arrow) represents the ROI containing representative areas of bone (blue) and background (green). Bar = 300 µm. C) Red area represents AI-identified osteoclasts stained for cathepsin K. Bar = 50 µm D) Dotted blue line on the very edge of the bone represents the interphase between ROI_Bone_ and ROI_Background_. Red area represents AI-identified osteoclasts stained for TRAP. Note that only AI-identified osteoclasts residing on the bone surface will be included since the interphase length between ROI_Bone_ and ROI_Background_ is used to calculate Oc.S/BS. Bar = 50 µm.
  E. Use the *Wheel* and select *ROI* or use the hotkey F4 to show the *ROI Drawing* menu. Click *Polygons or Freehand* and use the annotated lines as a guide to draw a new ROI_Default_. The ROI should be carefully delineated along the endosteal bone surface, so it encompasses trabecular bone only.
  F. For clarity, the annotated lines can be deleted under *Annotation Drawing → Draw → Delete* (red cross).

Step-by-step procedures for AI-assisted osteoclast identification and Oc.S/BS estimation using scanned sections. For clarity, the name of buttons or tabs in the VIS software is written in *italic*. The procedure is divided into the three overall steps 1. Annotation, 2. App-creation and algorithm training for the bone classifier, and 3. App-creation, algorithm training for classifiers, and calculation of Oc.S/BS:

1. Annotation:
  A. Right-click in the working area of VIS to activate the *Wheel* and select *Annotate*. Alternatively, use the hotkey F5 to display *Annotation Drawing*.
  B. Use *Poly Line* to annotate along the distal part of the growth plate of the proximal tibial metaphysis.
  C. Click *Measure* on the *Wheel* and use *Ruler* or use the hotkey F2 to display the measurement menu and find a point e.g. 1500 µm below the annotated growth zone line to exclude primary spongiosa from the region of interest (ROI). We suggest a perpendicular distance of 1500 µm below the annotated growth zone line in rats and 300 µm in mice.
  D. Copy the growth zone annotation line and paste it at the desired distance (e.g. 1500 µm) using the copy/paste function under *Annotation Drawing → Draw*. In addition, paste the growth zone annotation line again 3000 µm below the prior annotated line for rats and 1000 µm for mice (Fig. 2A).

2. App-creation and algorithm training for classifiers
  G. First, find representative areas of bone and background (e.g. bone marrow and artifacts) on one of the scanned sections.
  H. Create a ROI and roughly outline bone (blue) and background (green) using the Wheel and click Label (Figure 2B). Rename the green label to Background and the blue label to Bone.
    In theory, larger and more diverse training sets will improve the classification. Therefore, we recommended to repeat steps G and H on several ROIs and sections.
  I. Create an app that recognizes calcified bone. Click New App → New Class and select Background as the top class and Bone as the second class.
  J. Open the Classification tap, choose Deep Learning as method, and use U-Net (Default). Leave other settings at their default values.
  K. Under Input choose either 20x or 10x magnification.
    We suggest training and running the app at the highest magnification. However, the analysis time will be vastly reduced if *10x* is used.
  L. Under Regions To Analyze press Add/Remove Regions and choose ROI_Default_.
  M. Press the Train button.
  N. Visiopharm recommend running the app for 100,000 iterations; however, we suggest a minimum of 10,000 iterations in the first training run.
  O. Press Preview to preview the training. If the app is inconsistent or make misclassifications (bone as background or vice versa), repeat steps G and H and press Continue Training.
    App-training is an iterative process that have to be continued until an acceptable classification is achieved and might require several training runs.
  P. Use the Wheel on the image and select ROI and rename ROI 002 to ROI_Bone_.
  Q. Under Post Processing, click New Post Processing Step and add Outline As ROI. Under Label select Background and under To ROI select ROI_Background_.
  R. Under Post Processing click New Post Processing Step and add Outline As ROI. Under Label select Bone and under To ROI select ROI_Bone_.
  S. We suggest to add a post-processing step Close of 10–40 µm to smooth the interphase border in order to more precisely adhere to the natural border of the trabeculae. Under Post Processing, press New Post Processing Step to add Close. Small, misclassified areas can be removed using the post-processing step Change by Shape.
  T. Load the annotated slides to the Slide Tray and press Run on the app.
  U. Visual inspection and manual correction after the app have completed might be necessary to remove obvious misclassifications.

3. App-creation, algorithm training for osteoclast classifier, and calculation of Oc.S/BS
  V. Repeat step G–O. Replace Bone with Osteoclasts in step H–I and make it a third image classifier (red).
  W. Replace ROI_Background_ with ROI_Bone_ in step l.
    For an example of an osteoclast stained for cathepsin K or TRAP used as classifier see Figure 2C and 2D, respectively.
  X. Under Output Variable press New Variable.
  Y. Bone surface (BS) is defined as the length of the interface between ROI_Bone_ and ROI_Background_ Oc.S is defined as the length of the interface between the osteoclast area and ROI_Bone_ area. Oc.S/BS can now be calculated as an output variable.

## Method validation

**Fig. 3:**
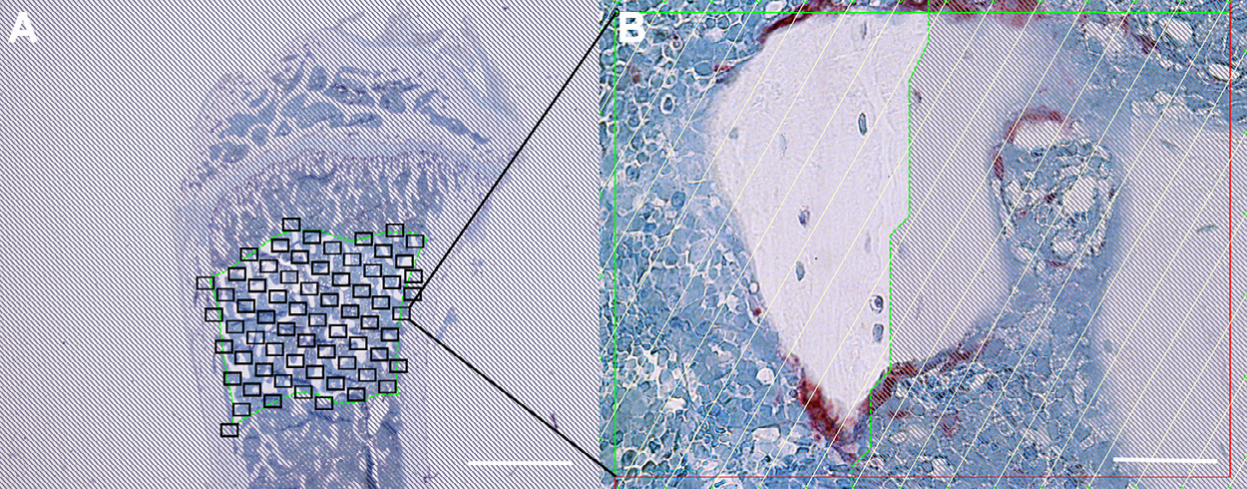
A) ROI at the proximal tibial metaphysis delineating the trabecular compartment used to sample sixty-five random positions covering 40% of the region of interest (ROI). Bar = 2 mm. B) Counting frame with superimposed grid within the ROI. Bar = 50 µm.

To verify the method, we compared AI-assisted osteoclast identification with conventional manual identification of osteoclasts using a bright-field microscope with the same ROIs as used for the AI-based analysis. A systematic uniform random sampling of the designated ROI covering at least 40% using the 40x lens at a final magnification of × 1132 was performed using a randomly rotated superimposed counting grid (Fig. 3A). Bone was defined as an intact bone surface intersecting directly with one or more lines of the counting grid. Osteoclasts were defined as TRAP or cathepsin-K positive cells, with one or more nuclei, located on an intact trabecular bone surface, which intersected one or more lines of the counting grid (Fig. 3B). To determine Oc.S/BS the total number of intersections with osteoclasts was divided by the total number of intersections with a bone surface. In addition, the time elapsed during osteoclast identification and counting was recorded.

### Statistics

Data were analyzed using a paired Student's *t*-test and a Bland-Altman plot. Results were defined as statistically significant if the two-tailed *p* < 0.05. Statistical analysis and graph drawing were conducted using GraphPad Prism 8.4 (GraphPad Software, San Diego, CA, USA). The Oc.S/BS results obtained with manual bright-field microscopy have previously partly been described [7,8], whereas the AI-assisted estimations are new.

### Results

**Fig. 4:**
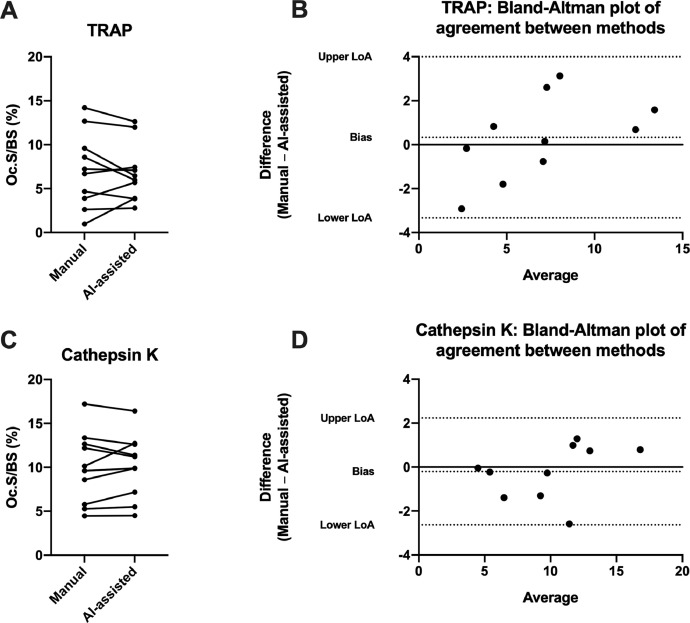
Connected plot illustrating the difference in estimated amount of osteoclast covered surfaces (Oc.S/BS) from analyzed bone samples using the manual and AI-assisted method. Bland-Altman plot of agreement between the manual and AI-assisted method. LoA: Limits of agreement (95%). A) and B) Results from sections stained for TRAP. C and D: Results using cathepsin K antibody.

Determination of Oc.S/BS using AI-assisted quantification were 60% faster than the manual quantification. Importantly, the Oc.S/BS estimates did not differ between the AI-assisted and manual method using either osteoclasts stained for TRAP (*p* = 0.59) or cathepsin K (*p* = 0.62) (Table 1). Using sections stained for TRAP, the AI-assisted method found that Oc.S/BS = 6.78%, while the manual method found that Oc.S/BS = 7.11% (Fig. 4A). Using sections stained for cathepsin K, the AI-assisted estimation found that Oc.S/BS = 10.13%, while the manual quantification found that Oc.S/BS = 9.93%. The two methods were compared using a Bland Altman plot (Fig. 4B). For sections stained for TRAP, the Bland-Altman plot showed a bias of 0.33 with 95% limits of agreement from −3.33 to 3.99. For sections stained for cathepsin K, the Bland-Altman plot showed a bias of −0.20 with 95% limits of agreement from −2.63 to 2.23.

**Table 1:** Average time used per sample and average amount of osteoclast covered surfaces (Oc.S/BS) from sections stained for TRAP or immunostained for cathepsin K. Time per sample data are from sections stained for TRAP. Data are presented as mean ± SD. * Denotes significant difference (*p* < 0.05) compared to Manual.

	Manual	AI-assisted
Time per sample (minutes)	16.2 ± 4.22	6.42 ± 1.66*
Oc.S/BS (%) using TRAP	7.11 ± 4.26	6.78 ± 3.28
Oc.S/BS (%) using Cathepsin K	9.92 ± 4.05	10.13 ± 3.60

## Strengths, limitations and future perspectives

The most obvious strength of using an AI-based identification and counting of osteoclasts such as the AI-module provided by Visiopharm is the ability to speed-up the tedious and repetitive process of manual identification and counting. Annotation is straightforward, easy to delegate, and does not require an in-depth understanding of advanced programming languages. Another advantage is the ability to run a full-automatic batch queue of multiple scanned sections overnight. Furthermore, retraining, when misclassifications occur, allows for an app that learns and therefore potentially increases in sensitivity over time.

Although deep learning classification has a huge potential to ease and speed-up trivial tasks like image processing, it is still limited by the quality of the histological sections used for algorithm training and subsequent analysis. Deep learning brings us closer to hands-off quantitative image analysis, however, manual corrections and inspection of results may still be needed in order to minimize misclassification.

Another limitation of the method is that the VIS software required for AI analysis is proprietary software.

## Conclusion

AI-assisted image processing for quantification of Oc.S/BS is less time consuming than manual quantification using a bright-field microscope and provides results that did not differ from those obtained by the conventional method.
